# Genetic Spectrum and Cascade Screening of Familial Hypercholesterolemia in Routine Clinical Setting in Hong Kong

**DOI:** 10.3390/genes14112071

**Published:** 2023-11-13

**Authors:** Man-Kwan Yip, Elaine Yin-Wah Kwan, Jenny Yin-Yan Leung, Emmy Yuen-Fun Lau, Wing-Tat Poon

**Affiliations:** 1 Department of Clinical Pathology, Pamela Youde Nethersole Eastern Hospital, Chai Wan, Hong Kong, China; 2 Department of Paediatrics and Adolescent Medicine, Pamela Youde Nethersole Eastern Hospital, Chai Wan, Hong Kong, China; elaine.kwan@ha.org.hk; 3 Department of Medicine and Geriatrics, Ruttonjee Hospital, Wan Chai, Hong Kong, China; leungyyj@ha.org.hk; 4 Department of Medicine, Pamela Youde Nethersole Eastern Hospital, Chai Wan, Hong Kong, China

**Keywords:** familial hypercholesterolemia, genetic spectrum, cascade screening, *LDLR* gene

## Abstract

Familial hypercholesterolemia (FH) is a prevalent but often underdiagnosed monogenic disorder affecting lipoprotein metabolism, and genetic testing for FH has not been widely conducted in Asia in the past. In this cross-sectional study of 31 probands (19 adults and 12 children) and an addition of 15 individuals (12 adults and 3 children), who underwent genetic testing and cascade screening for FH, respectively, during the period between February 2015 and July 2023, we identified a total of 25 distinct *LDLR* variants in 71.0% unrelated probands. Among the adult proband cohort, a higher proportion of genetically confirmed cases exhibited a positive family history of premature cardiovascular disease. Treatment intensity required to achieve an approximate 50% reduction in pretreatment low-density lipoprotein cholesterol (LDL-C) exhibited potentially better diagnostic performance compared to pretreatment LDL-C levels, Dutch Lipid Clinic Network Diagnostic Criteria (DLCNC) score, and modified DLCNC score. Adult individuals identified through cascade screening demonstrated less severe phenotypes, and fewer of them met previously proposed local criteria for FH genetic testing compared to the probands, indicating that cascade screening played a crucial role in the early detection of new cases that might otherwise have gone undiagnosed. These findings underscore the significance of genetic testing and cascade screening in the accurate identification and management of FH cases.

## 1. Introduction

Familial hypercholesterolemia (FH) is a highly prevalent inherited disorder, affecting approximately 1 in 200–500 individuals [1,2]. It is characterized by elevated levels of low-density lipoprotein cholesterol (LDL-C) and increased susceptibility to premature atherosclerotic cardiovascular disease (pCVD). Unfortunately, FH remains underdiagnosed and undertreated in many Asian countries [3]. In Hong Kong, for instance, the estimated percentage of individuals diagnosed with FH is a mere 2.2%, significantly lower than the 10–20% observed in the United Kingdom [4].

The lack of awareness and knowledge about FH among physicians across Asia further contributes to the challenge [5]. This highlights the pressing need for the recent Global Call to Action by the Global Familial Hypercholesterolemia Community, which advocates for early diagnosis and treatment of FH. Through increased awareness and proactive management, we can effectively improve prognosis and reduce cardiovascular morbidity and mortality associated with FH [6].

Diagnosing FH typically relies on clinical features rather than genetic testing, especially in resource-deprived countries where genetic testing may not be readily available. Various sets of clinical criteria have been proposed internationally and locally (Table 1), including the Simon Broome Register diagnostic criteria, the Make Early Diagnosis to Prevent Early Deaths (MEDPED) criteria, the Dutch Lipid Clinic Network Diagnostic Criteria (DLCNC), the modified Dutch Lipid Clinic Network Diagnostic Criteria for Chinese (modified DLCNC), and the Japanese FH Management Criteria (JFHMC). However, there is no universally accepted gold standard for diagnosing FH, and in 2018, a local guideline was proposed by an expert panel [7].

Monogenic FH is primarily caused by variants in the LDL receptor (*LDLR*) gene, the apolipoprotein B100 (*APOB*) gene, and the proprotein convertase subtilisin/kexin type 9 (*PCSK9*) gene. The spectrum of FH variants varies among different countries and ethnic groups. In Chinese populations, the majority of FH variants are found in the *LDLR* gene. Specifically, variants such as c.986 G>A, c.1448 G>A, c.1747 C>T, c.1879 G>A, and c.268 G>A have been reported as the most frequent in Chinese individuals [8,9,10,11].

Three main screening strategies have been proposed to assist in identifying individuals with FH and ensuring early intervention, including opportunistic, universal, and cascade screening. In most developed countries, such as Australia, Canada, and Spain, the most commonly employed method is opportunistic screening by detecting community cholesterol levels in primary healthcare services. On the other hand, universal screening of plasma cholesterol levels in children has been proposed in Australia, Canada, the United States, Slovenia (for preschool children aged 5–6 only), and Hong Kong. All published scientific statements and guidelines have recommended active case finding combined with family-based cascade screening [12], which has been adopted in routine healthcare settings in two local hospitals, with the utilization of genetic testing.

The objective of this study was threefold: first, to investigate the range of genetic variants present in individuals with FH in Hong Kong; second, to assess the diagnostic accuracy of different clinical diagnostic criteria proposed for FH; and third, to evaluate the effectiveness of genetic cascade screening. By addressing these objectives, we aimed to enhance our understanding of the genetic landscape of FH, improve diagnostic approaches, and determine the impact of cascade screening in identifying individuals at risk of FH.

**Table 1:** Comparison of diagnostic criteria for familial hypercholesterolemia (FH) [7,13].

Criteria	Simon Broome Register	MEDPED	DLCNC	Modified DLCNC	JFHMC	Hong Kong Expert Panel Recommendation
**Family history of pCVD or hyperlipidemia**	Y	Y	Y	Y	Y	Optional
**History of pCVD**	N	N	Y	Y	N	N
**Physical signs (e.g., tendon xanthoma)**	Y	N	Y	Y	Y	Optional
**LDL-C cutoff (mmol/L)**	Adult: >4.9 Children: > 4.0	By total cholesterol: specific levels based on individual’s age and a family history of FH	• ≥8.5: 8 points • 6.5–8.4: 5 points • 5.0–6.4: 3 points • 4.0–4.9: 1 point	• ≥6: 8 points • 5–5.9: 5 points • 3.5–4.9: 3 points • 2.5–3.4: 1 point	For HeFH: Adult: >4.7 Children: >3.6 For HoFH: Total cholesterol: >15.5 mmol/L	Adult: >5; >4.5 if with family history of FH or pCVD Children: >3.6 if with family history; >4.9 and/or physical signs (e.g., xanthomata)
**Genetic study**	Optional	N	Optional	Optional	N	Optional
**Diagnosis**	• Definite FH • Possible FH	With FH	• Definite FH • Probable FH • Possible FH	• Definite FH • Probable FH • Possible FH	With FH	With FH
**Merit**	• Higher specificity than MEDPED • Ease of remembrance • Economic viability	• Higher sensitivity than DLCNC and Simon Broome Register criteria • Easy to use	• Higher specificity than MEDPED • Each criterion is weighted • Molecular defect leading to FH addressed	• Higher sensitivity than DLCNC	• High specificity and sensitivity in Japanese population	• Not evaluated so far
**Demerit**	• Lower sensitivity than MEDPED • Cannot discriminate between FH and secondary causes	• Lower specificity than DLCNC and Simon Broome Register criteria • Without regard to physical symptoms and a history of pCVD	• Lower sensitivity than MEDPED • Not applicable to children • Lack of versatility of use	• Lower specificity than DLCNC		

Y: Yes; N: No; MEDPED: Make Early Diagnosis to Prevent Early Deaths; DLCNC: Dutch Lipid Clinic Network Diagnostic Criteria; JFHMC: Japanese FH Management Criteria; LDL-C: low-density lipoprotein cholesterol; pCVD: premature atherosclerotic cardiovascular disease; HeFH: heterozygous FH; HoFH: homozygous FH.

## 2. Materials and Methods

We conducted a cross-sectional study involving unrelated index patients diagnosed with FH or severe hypercholesterolemia at two regional hospitals in Hong Kong between February 2015 and July 2023. These patients were identified from the clinic database. A comprehensive clinical assessment was performed to exclude secondary causes of hypercholesterolemia. Individuals found to have alternative genetic diagnoses in subsequent genetic testing were excluded from this study. Written informed consent had been obtained from all patients, and the study was approved by the Hong Kong East Cluster Research Ethics Committee of the Hospital Authority, Hong Kong (approval number HKEC-2016-065).

The diagnosis of FH was retrospectively categorized as definite, probable, or possible FH based on the Dutch Lipid Clinic Network Criteria (DLCNC) and the modified DLCN Criteria for Chinese [14]. The modified DLCN Criteria for Chinese utilizes lower LDL-C cutoffs specific to the Chinese population and has been reported to have higher sensitivity but lower specificity [15].

Pretreatment lipid levels were available in the medical records of 74.0% of the index patients. For those without pretreatment LDL-C data, we estimated the untreated LDL-C levels using correction factors for statins and ezetimibe treatment [16]. Furthermore, we translated these correction factors into treatment intensity measures for the patients (Table S1).

Genetic analysis was performed on the index patients, and their first-degree relatives (parents, offspring, and siblings) were invited to participate in cascade screening. During cascade screening, targeted testing was conducted on the familial variant to identify potential new cases within the family. Plasma lipid levels were measured in all family members who underwent screening. Total cholesterol, high-density lipoprotein cholesterol (HDL-C), and triglyceride levels were measured using standardized enzymatic methods on the Abbott Architect c16000 system, while LDL-C concentrations were calculated using the Friedewald formula. LDL-C would not be reported if the triglyceride level was greater than 4.5 mmol/L, and the highest LDL-C ever available for the patient would be included in our analysis instead. All the assays had passed external quality assessment and internal quality control prior to the reporting of results and had been accredited by the Hong Kong Laboratory Accreditation Scheme.

Genetic analyses for all coding exons and respective 10-base pair flanking regions of *APOB*, *LDLR*, and *PCSK9* genes were performed. Sanger sequencing was performed on 9 adults and 8 probands and all cascade screening subjects, while next-generation sequencing (NGS) was performed on 10 adults and 4 children probands. For Sanger sequencing, DNA from peripheral blood was extracted using Qiagen QIAamp DNA Blood Mini Kit (Qiagen, Hilden, Germany) following the manufacturer’s instructions. Target exons were amplified from extracted genomic DNA by PCR. Sanger sequencing was performed using the BigDye Terminator v1.1 Cycle Sequencing Kit (Applied Biosystems, Foster City, CA, USA) and an ABI 3500 genetic analyzer. For NGS, DNA extraction and purification were performed on the submitted sample using Qiagen QIAamp DNA Blood Mini Kit (Qiagen, Hilden, Germany) and enriched using the MGIEasy Exome Rapid Library Prep Kit/Exome Capture V5 Universal Kit (MGI Tech, Shenzhen, China) according to the manufacturer’s instructions. One hundred-base pair, paired-end (PE100) DNA sequencing was performed on a BGISEQ-500 sequencer (MGI Tech, Shenzhen, China). Variant calling and filtering were performed using an in-house bioinformatics pipeline. In general, all target regions were sequenced with 20× or greater coverage with a Phred-scaled quality score of 20 or above, and a mapping quality score of 20 or above, and quality-checked using SAMtools (version 0.3.3) [17] and a custom in-house Python script. Exceptionally, target regions with sequencing coverage below the quality standard would be individually sequenced using Sanger sequencing.

Sequence analysis could detect around 85%, 100%, and 98% of pathogenic variants in *LDLR*, *APOB*, and *PCSK9* genes, respectively, which account for around 60% of known genetic causes of FH [18].

For statistical analysis, numeric data were compared by independent sample t-test or Mann–Whitney U test, and proportions were compared by Fisher’s exact test. *p*-value < 0.05 (two-tailed) was considered as significant. Data analysis was performed using IBM SPSS Statistics (version 23.0.0.0). Receiver operating characteristic (ROC) curve analysis was performed using MedCalc (version 22.006).

## 3. Results

### 3.1. Genetic Spectrum Identified in the Entire Cohort

This study included 31 probands (19 adults and 12 children) undergoing genetic testing for FH, as well as 15 individuals (12 adults and 3 children) undergoing cascade screening with genetic testing. Three adult probands (1 met all the clinical criteria for FH and 2 only met the MEDPED criteria) were found to have alternative genetic diagnoses subsequently and were excluded from this study. All newly diagnosed FH patients received counseling and were scheduled for follow-up. In the adult cohort, genetic analysis revealed that 14 subjects had heterozygous FH, and 2 subjects had compound heterozygous FH. In the pediatric cohort, 4 cases of heterozygous FH and 2 cases of compound heterozygous FH were detected. Variants were identified in 84.2% of adult probands (*n* = 16) and 50.0% of child probands (*n* = 6), all affecting the *LDLR* gene. A total of 25 distinct variants were identified in 71.0% of unrelated index FH cases (22 out of 31 probands). All the identified variants in this study were classified as expected receptor-negative or receptor-defective variants based on variant types [19] in Table 2 and Table 3. The two most common variants in the *LDLR* gene were c.1241 T>G and c.986 G>A. Fourteen newly identified genetic variants in the *LDLR* gene were found in our adult and pediatric cohorts compared to previous local studies [20,21,22], expanding the genetic spectrum of *LDLR*-related FH observed locally by 23.7%.

In this cohort, two novel variants were identified. One compound heterozygous FH subject carried the novel likely pathogenic c.837 dupC frameshift variant along with the known c.1247 G>A missense variant. This subject had a baseline LDL-C of 8.8 mmol/L and a normal phytosterol profile. The diagnosis was made at the age of 6 due to the presence of tendon xanthoma, and there was a strong family history of hyperlipidemia in multiple generations, including the father, paternal grandmother, mother, and maternal grandmother. The c.1247 G>A missense variant was detected in the subject’s mother, who had a pretreatment LDL-C of 3.86 mmol/L. However, the c.837 dupC frameshift variant was not found in either the father or mother, suggesting the possibility of germline mosaicism in one of the parents or a de novo variant in the subject. This variant was predicted to introduce a premature stop codon downstream of the duplication site, resulting in a truncated LDLR protein. The patient, currently 10 years old, was on a daily regimen of Rosuvastatin 20 mg and Ezetimibe 10 mg. The latest LDL-C measurement was 3.01 mmol/L, and a complete resolution of xanthoma has been achieved.

Additionally, another novel splice donor variant, c.1060+2 T>C, was detected in an adult heterozygous FH patient who presented with incidentally elevated LDL-C of 8.3 mmol/L at the age of 38. He had a strong family history of hyperlipidemia, with his father experiencing pCVD in his 50s. The patient is currently on Rosuvastatin 20 mg and Ezetimibe 10 mg daily, and his latest LDL-C measurement was 3.87 mmol/L.

### 3.2. Findings in the Adult Cohort

In Table S2, the clinical profiles and pretreatment lipid levels of the adult probands and all adult individuals (including both probands and individuals undergoing cascade screening) are summarized. The median time gap between the clinical diagnosis of hyperlipidemia and the performance of genetic testing for FH in the probands was 18 years (interquartile range: 11 years). All probands fulfilled the DLCNC FH criteria (68.4%, 21.1%, and 10.5% of cases for definite, probable, and possible FH, respectively) and the criteria for genetic testing of FH as recommended by a local expert panel. Approximately 95% of the probands had a positive family history of hyperlipidemia in their first-degree relatives, and 58% of them were overweight or obese. Physical signs of hypercholesterolemia were not commonly observed in this cohort, and only around 20% of probands were noted to have xanthoma or xanthelasma. The mean pretreatment total cholesterol and LDL-C were 10.28 and 8.42 mmol/L, respectively. The mean DLCNC score and modified DLCNC score were 9.4 and 10.5, respectively. All probands were being treated with statins, with or without ezetimibe, and 21.1% were currently receiving PCSK9 inhibitors. However, only around 32% and 11% of patients were able to achieve the target LDL-C level of less than 2.5 mmol/L and 1.8 mmol/L, respectively.

Table S3 provides a summary of the clinical profiles and pretreatment lipid levels of the adult probands with positive and negative genetic results. There were no significant differences observed between the two groups in terms of pretreatment total cholesterol (10.35 vs. 10.00 mmol/L, *p* = 0.836), pretreatment LDL-C levels (8.77 vs. 6.53 mmol/L, *p* = 0.137), peak LDL-C levels (9.24 vs. 10.50 mmol/L, *p* = 0.244), and DLCNC scores (9.8 vs. 7.3, *p* = 0.254). This indicates a high overlap in clinical features between the two groups in this cohort and highlights the difficulty in diagnosing FH based solely on clinical criteria. However, it was noted that the genetically confirmed group had a higher rate of positive family history of pCVD (75.0% vs. 0.0% *p* = 0.036). Additionally, the peak LDL-C level was found to be higher than the pretreatment LDL-C level in the genetically confirmed group (9.24 vs. 8.77 mmol/L, *p* = 0.041), suggesting that the pretreatment LDL-C level might not represent the highest value reached by the patient throughout their lifetime, leading to a delayed suspicion of FH later in life.

When considering all adult individuals, regardless of whether they were probands or individuals identified through cascade screening, those with a positive genetic diagnosis had a higher rate of positive family history of pCVD (69.2% vs. 0.0%, *p* = 0.008) and were currently receiving more intensive treatment (2.56 vs. 0.86, *p* = 0.007) to achieve a reduction in LDL-C levels by approximately 50% (55.0% vs. 55.5%, *p* = 0.967). However, the difference in LDL-C levels between the two groups (7.94 vs. 5.59 mmol/L, *p* = 0.050) was only marginally significant.

After identifying the adult probands, a total of 12 first-degree relatives underwent screening, and 10 of them were found to have FH. Adult individuals identified through cascade screening had significantly lower pretreatment total cholesterol levels compared to the probands (8.13 vs. 10.35 mmol/L, *p* = 0.025). They also had lower pretreatment LDL-C levels (6.60 vs. 8.77 mmol/L, *p* = 0.047) and peak LDL-C levels (6.77 vs. 9.24 mmol/L, *p* = 0.008). Furthermore, the DLCNC score (5.8 vs. 9.8, *p* = 0.003) and modified DLCNC score (7.8 vs. 10.8, *p* = 0.014) were lower in the individuals identified through cascade screening compared to the probands. A smaller proportion of individuals identified through cascade screening met the previously proposed local criteria for FH genetic testing compared to the probands (70.0% vs. 100.0%, *p* = 0.046). These findings suggest that cascade screening plays a significant role in the early detection of new FH cases that may otherwise be missed. (These results were not shown in tables in this article.)

**Figure 1:**
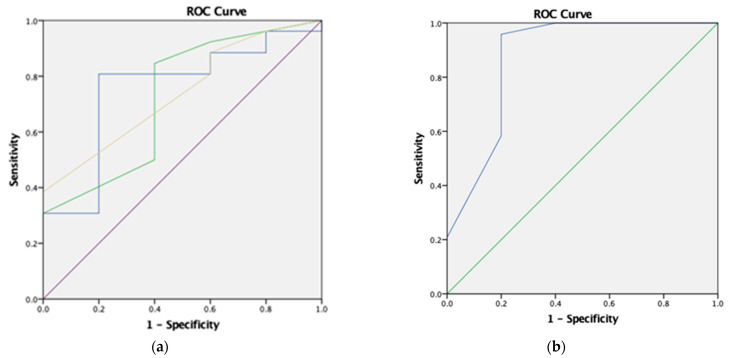
(**a**) ROC generated by pretreatment LDL-C (blue), DLCNC score (green), and modified DLCNC score (yellow) (AUC = 0.754, 0.723, and 0.738, respectively); (**b**) ROC generated by treatment intensity yielding an around 50% drop of pretreatment LDL-C (AUC = 0.875).

To evaluate the optimal pretreatment LDL-C cutoff level to predict the presence of pathogenic variant(s) in all adult subjects screened in this highly selective cohort, an ROC curve was generated and the point with the maximum Youden index (J = sensitivity + specificity − 1) was determined on the ROC curve (Figure 1a). The area under the ROC curve (AUC) was 0.754, and the optimal pretreatment LDL-C cutoff level obtained using the Youden index was 6.32 mmol/L, with a sensitivity of 80.8% and specificity of 80.0%. The AUC generated by the DLCNC score and modified DLCNC score were 0.723 and 0.738, respectively, with the optimal DLCNC score and modified DLCNC score obtained using Youden index being 5.0 (sensitivity of 84.6% and specificity of 60.0%) and 9.5 (sensitivity of 38.5% and specificity of 100.0%) for the diagnosis of genetically confirmed FH in our study population, in contrast to the cutoff of 3 established previously. In addition, it was found that the optimal cutoff for treatment intensity yielding around 50% drop of pretreatment LDL-C for the correct diagnosis of FH was 1.8 (sensitivity of 95.8% and specificity of 80.0%), corresponding to the use of moderate-intensity statin with dosage equivalent to Simvastatin 40 to 80 mg daily (treatment intensity of 1.7 to 1.9), Atorvastatin 20 mg daily, or Rosuvastatin 5 mg daily. The AUC of this treatment intensity measure was 0.875, greater than that generated by pretreatment LDL-C, DLCNC score, or modified DLCNC score (Figure 1b), although not reaching statistical significance probably due to the small sample size. However, when compared with the diagnostic performance of the established cutoff of 3 for both DLCNC and modified DLCNC scores, which all probands had met, the treatment intensity measure still outperformed.

The diagnostic performance of Simon Broom Register criteria, MEDPED, JFHMC, a recommendation made by a local expert, as well as pretreatment LDL-C cutoff of 5.5 mmol/L previously suggested by a local study were assessed against the genetic results (Table 4), which all showed a sensitivity greater than 80% but a poor specificity in our adult cohort. Combining the criteria of treatment intensity yielding an around 50% drop of pretreatment LDL-C ≥ 1.8 and LDL-C cutoff of 5.5 mmol/L, which was found to be the optimal cutoff in a previous local study [20], the specificity could increase to 100.0%, but with a lower sensitivity of 83.3% when compared to using treatment intensity as the sole criteria.

### 3.3. Findings in the Pediatric Cohort

Considering all the 15 pediatric individuals consisting of both probands and individuals identified through cascade screening, the mean ages of diagnosis of hyperlipidemia and FH were 8.6 and 9.8, respectively. All of them met the clinical criteria of FH according to Simon Broome Register, MEDPED, JFHMC, and the local expert panel, while their mean pretreatment LDL-C was 6.88 mmol/L (Table S4). Six out of twelve probands and all three individuals identified through cascade screening were found to have FH. The mean DLCNC scores were 8.2 and 1.8 for genetically positive and negative index cases, respectively (Table S5). DLCNC score is not validated and may cause under-diagnosis without using LDL-C cutoffs specific to the pediatric population [23,24]. This might explain why the negative index cases would have a DLCNC score lower than 3 while fulfilling all other clinical criteria of FH.

## 4. Discussion

**Figure 2:**
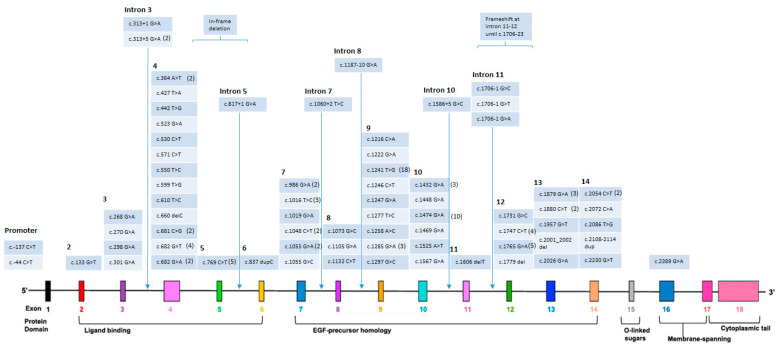
73 variants identified in Hong Kong FH subjects [20,21,22]. Frequencies of variants detected were marked in brackets if more than one.

In this cohort of clinically diagnosed FH patients in Hong Kong, our findings reveal that more than 70% of the patients had a genetic basis, with all identified causative variants located in the *LDLR* gene. In Hong Kong, a total of 73 different *LDLR* variants have been reported (Figure 2), which is fewer than the 143 different *LDLR* variants identified in FH subjects of Han Chinese descent in a recent review [10]. The most common variants in the *LDLR* gene in Hong Kong were c.1241 T>G (13.8%), c.1474 G>A (7.7%), c.769 C>T (3.8%), and c.1765 G>A (3.8%). Notably, the c.1241 T>G variant is uncommon in Western populations [20]. Consistent with previous findings [9], the majority of mutations were located in exon 4 and exon 9.

Within our study, two novel variants in the *LDLR* gene were identified, and their pathogenicity was predicted through in silico analysis. However, we did not detect any variants in the *APOB* gene or *PCSK9* gene in our cohort, which is also uncommon based on a previous local study [20]. Typically, in most populations, *PCSK9* gene variants are found in less than 1% of FH subjects [15,20]. This is supported by a recent large-scale study, which identified variants in the *PCSK9* gene associated with FH in only 0.6% of over 26,000 individuals [25].

The existing clinical criteria demonstrated good sensitivity in identifying patients with FH in Hong Kong; however, their specificity was questionable, potentially leading to an increased rate of unnecessary genetic testing. This finding is consistent with previous local research (Table 5). In our cohort, the optimal pretreatment LDL-C cutoff level for predicting the genetic diagnosis of FH was determined to be 6.32 mmol/L. This value is higher than the previously reported cutoff of 5.5 mmol/L in Hong Kong [20], likely due to the higher LDL-C levels in our study participants.

Despite treatment with high-intensity statins, only a few FH subjects were able to achieve the recommended LDL-C goal [26]. In our study, we attempted to evaluate the cutoff for treatment intensity that would result in approximately a 50% reduction in pretreatment LDL-C, aiming to predict genetically confirmed FH. Although statistically significant differences in AUCs between this approach and other existing quantitative criteria had not yet been demonstrated, this indicator may potentially serve as a simpler tool for clinicians to consider FH in hyperlipidemia patients who do not respond optimally to treatment. However, it is important to note that the intensity of treatment could be influenced by statin intolerance, and cholesterol response varies based on adherence and racial differences [27]. Further research on this measure is therefore highly recommended.

Studies have suggested that lower statin doses can achieve lipid improvements in Asian patients comparable to higher doses in Caucasians [28] and may explain why the observed treatment intensity cutoff to achieve the desired LDL-C reduction by around 50% fell within the moderate intensity range. This level of treatment intensity was expected to lower LDL-C by 30% to <50% in Western populations [29].

In our cohort, the presence of a positive family history of pCVD was 100% specific to genetically confirmed FH. This finding is similar to the high prevalence of family history of pCVD with a less frequent personal history observed in a Spanish cohort of patients with a genetic diagnosis of FH [30]. As the current scoring system assigns only one point for either the presence of a positive history of pCVD or hyperlipidemia in a first-degree relative, considering the presence of a positive family history of pCVD separately from hyperlipidemia may increase its weighting due to its high specificity. However, further evaluation in the local population is needed to validate this approach.

The availability of genetic testing has been identified as a barrier to the early diagnosis and management of FH [31]. Genetic testing for FH offers several benefits, including providing a definitive molecular diagnosis, offering prognostic information, enabling refined CVD risk stratification, and promoting treatment initiation and compliance, eventually leading to LDL-C reduction. Recognizing these clinical benefits, the NICE (National Institute for Health and Care Excellence) guidelines and the European Society of Cardiology (ESC) and European Atherosclerosis Society (EAS) guidelines emphasize the importance of confirming the diagnosis of FH through genetic testing and performing cascade genetic screening [32]. Cascade genetic screening has the potential to identify up to eight additional FH cases per proband and has been designated a “Tier 1 Genomics Application” by the Centers for Disease Control and Prevention’s (CDC) Office of Genomics and Precision Public Health [33]. Studies have shown that genetic cascade screening for FH is more cost-effective compared to no screening, with the incremental cost-effectiveness ratios (ICERs) reported to be 29,608 EUR per quality-adjusted life years (QALY) and 8700 USD per life year gain. However, no studies comparing the cost-effectiveness of genetic cascade screening and lipid cascade screening were conducted after 2015; therefore, further research would be warranted due to the decreasing cost of genetic testing nowadays [12,34,35].

Our study demonstrates that this strategy enables the early detection of new FH cases that might otherwise be missed. By identifying these cases early, more intensive treatment can be initiated as soon as possible, even before the development of coronary artery disease. In our adult cohort, the time delay in confirming the genetic diagnosis of FH was 14 years, which was shorter than the reported delay of over 18 years in the Spanish population [30]. This highlights the efforts made by our local healthcare team in the early recognition of FH. However, it is important to note that earlier detection would always be preferable in order to optimize patient outcomes.

This study had several limitations that should be acknowledged. Firstly, the patients referred for genetic testing in our study mainly had severe phenotypes, which might not be representative of the entire FH population. Additionally, the sample size was relatively small, which could limit the generalizability of our findings. It is important to conduct further research with larger and more diverse cohorts to validate our results.

Moreover, cascade genetic screening is reliant on systematic family tracing as well as the prior effective index case identification, which can be accomplished through universal screening of children or young adults, or opportunistic screening of adults with a family history of pCVD or hypercholesterolemia in primary care. The key to the success of the national genetic cascade screening program in the Netherlands, which identified over 70% of all individuals with FH nationwide, is postulated to be the presence of a government-funded centralized coordinating office, which facilitated direct contact with relatives and in-home visits for sample collection [33]. In our study, most of our index cases were identified during specialist clinic follow-up or hospital admission, and probands would need to contact their first-degree relatives by themselves for cascade screening. The cost-effectiveness of cascade screening combined with different index case identification and family tracing strategies should be further evaluated.

Approximately 30% of our patients did not have a detectable genetic etiology, which is consistent with previous studies. We should note that our genetic screening did not include the analysis of large deletions or insertions in the *LDLR* gene [36] and some other gene variants associated with the FH phenotype, such as *LDLRAP1*, *ABCG5*, *ABCG8*, *CYP27A1*, *LIPA*, and *LIPG* [37]. The frequency of large *LDLR* rearrangements in Taiwanese FH patients was reported to be around 8% [38]. Furthermore, there might be unknown monogenic or polygenic causes of hypercholesterolemia contributing to the observed phenotypes in some of our subjects [20].

Three probands with alternative genetic diagnoses related to hyperlipidemia had been excluded from this study. One case involved a suspected sitosterolemia patient who presented with pCVD at the age of 35, despite being an active marathon runner. Two cases were identified with hypertriglyceridemia associated with a homozygous *APOA5* pathogenic variant. These findings highlight the importance of considering alternative genetic diagnoses before reaching a clinical diagnosis of FH since the treatment and prognosis for these conditions often differ from FH.

It is worth noting that the patient with suspected sitosterolemia had specific genetic variants detected, including a variant of uncertain significance. While the patient was on rosuvastatin and ezetimibe therapy, his LDL-C levels remained high due to poor drug compliance. It is important to recognize that statin therapy, which is the first-line treatment for FH, is not typically indicated for the treatment of sitosterolemia unless atherosclerosis is present, and it does not lower plant sterol serum levels [39].

Regarding the two cases of hypertriglyceridemia associated with a homozygous *APOA5* pathogenic variant, both patients lacked a family history of hyperlipidemia. One study indicated that individuals carrying this allele had a significantly higher risk of developing hypertriglyceridemia [40]. While FH is typically characterized by normal HDL-C and triglyceride levels, it is important to consider that hypertriglyceridemia can coexist with FH [37]. However, it is worth noting that in the Wales Familial Hypercholesterolemia Service criteria, which is a modified version of the DLCNC, the presence of high fasting triglycerides is assigned a negative score [16].

Lastly, it is important to acknowledge that the Friedewald equation used to estimate LDL-C levels has been reported to underestimate LDL-C, particularly as triglyceride levels increase, potentially leading to misclassification of patients into incorrect cardiac risk categories [41]. This highlights the need for caution when interpreting LDL-C values based on the Friedewald equation.

These findings underscore the importance of considering alternative genetic diagnoses before reaching a clinical diagnosis of FH, as the treatment and prognosis for these conditions often differ from FH. Further research is warranted to explore the genetic causes of FH and other related hyperlipidemias, as well as to refine diagnostic criteria and improve patient management.

## 5. Conclusions

In summary, the majority of identified causative variants in individuals with FH in Hong Kong have been found in the *LDLR* gene, with the most common variant being NM_000527.5(*LDLR*): c.1241 T>G. The currently used clinical criteria have shown low specificity in identifying genetically confirmed FH cases in Hong Kong. A potential alternative approach could be to consider the treatment intensity required to achieve a significant reduction in LDL-C levels as a clinical criterion for identifying adult individuals with FH.

Genetic cascade screening has proven valuable in detecting genetically confirmed FH in family members who may exhibit less severe phenotypes, which might otherwise go undetected in routine clinical practice. It is important to consider the possibility of sitosterolemia and hypertriglyceridemia related to the *APOA5* gene, as these conditions can present with high cholesterol levels in adults. Proper evaluation and diagnosis of these conditions are crucial, as their management differs from that of FH.

## Figures and Tables

**Table 2:** Variants identified in this cohort categorized by variant types.

Types of *LDLR* Variants	Total Number of Cases	Adult	Pediatric	Total Number of Unique Variants	Newly Reported in Hong Kong Population	Total Number of Novel Variants
All	22	16	6	25	14	2
(1) Single variant	18	14	4	17	10	1
Receptor-negative	6	5	1	7	6	1
Splicing	3	3	0	3	3	1
Frameshift	1	1	0	1	1	0
Nonsense	2	1	1	2	2	0
Receptor-defective	12	9	3	10	4	0
Missense	12	9	3	10	4	0
(2) Two variants	4	2	2	8	4	1
Defective + Negative	2	0	2	4	3	1
Defective + Defective	2	2	0	4	1	0

**Table 3:** Details of individual variants identified in this cohort.

Variant	Protein Change	Localization	No. of Probands (No. by Cascade Screening)	Clinical Significance
*LDLR* NM_000527.5:				
c.268 G>A	p.(Asp90Asn)	3	1	Pathogenic
c.301 G>A	p.(Glu101Lys)	3	1	Pathogenic
c.523 G>A	p.(Asp175Asn)	4	1	Pathogenic
c.769 C>T	p.(Arg257Trp)	5	1	VUS
c.837 dupC	p.(Asn280GlnfsTer21) #	6	1	Likely pathogenic
c.986 G>A	p.(Cys329Tyr)	7	2 (1)	Likely pathogenic
c.1055 G>A	p.(Cys352Tyr)	7	1	Pathogenic
c.1060+2 T>C	Splice donor variant #	Intron 7	1	Pathogenic
c.1216 C>A	p.(Arg406=) New splice acceptor introduced	9	1	Likely pathogenic
c.1241 T>G	p.(Leu414Arg)	9	3 (3)	Likely pathogenic
c.1247 G>A	p.(Arg416Gln)	9	1 (1)	Pathogenic
c.1285 G>A	p.(Val429Met)	9	1	Pathogenic
c.1297 G>C	p.(Asp433His)	9	1	Likely pathogenic
c.1448 G>A	p.(Trp483Ter)	10	1 (2)	Pathogenic
c.1469 G>A	p.(Trp490Ter)	10	1	Pathogenic
c.1586+5 G>C	Intron variant	Intron 10	1	Likely pathogenic
c.1706-1 G>C	Splice acceptor variant	Intron 11	1	Pathogenic
c.1731 G>C	p.(Trp577Cys)	12	1 (4)	Pathogenic
c.1765 G>A	p.(Asp589Asn)	12	1 (1)	VUS/Likely pathogenic
c.1880 C>T	p.(Ala627Val)	13	1 (1)	Likely pathogenic
c.2001_2002 del	p.(Cys667*)	13	1	Pathogenic
c.2026 G>A	p.(Gly676Ser)	13	1	Likely pathogenic

# Novel variant. VUS: variant of uncertain significance.

**Table 4:** Diagnostic performance of various criteria suggested by international or local studies in the adult cohort.

Criteria (Prevalence = 84.2%)	Sensitivity	Specificity	PPV	NPV	Accuracy	*p*-Value
(1) Simon Broome Register	84.6%	40.0%	88.0%	33.3%	77.4%	0.241
(2) MEDPED	84.6%	40.0%	88.0%	33.3%	77.4%	0.241
(3) JFHMC	84.6%	40.0%	88.0%	33.3%	77.4%	0.241
(4) Hong Kong guideline	88.5%	20.0%	85.2%	25.0%	77.4%	0.525
(5) Pretreatment LDL-C ≥ 5.5 mmol/L	80.8%	60.0%	91.3%	37.5%	77.4%	0.093
(6) Treatment intensity ≥ 1.8 to achieve a drop of pretreatment LDL-C by around 50%	95.8%	80.0%	95.8%	80.0%	93.1%	0.001 *
(7) Items 5 and 6	83.3%	100.0%	100.0%	55.6%	86.2%	0.001 *

MEDPED: Make Early Diagnosis to Prevent Early Deaths; JFHMC: Japanese FH Management Criteria; LDL-C: low-density lipoprotein cholesterol. *: Statistically significant.

**Table 5:** Diagnostic performance of various criteria suggested by international studies if applied in Hong Kong adult cohort.

Criteria	Article	Sensitivity	Specificity
(1) Simon Broome Register	This study	84.6%	40.0%
	[15]	64.0%	56.6%
(2) DLCNC (cutoff of 3)	This study	100.0%	0.0%
	[15]	82.8%	53.3%
(3) Modified DLCNC (cutoff of 3)	This study	100.0%	0.0%
	[15]	93.8%	26.7%

## Data Availability

Data are contained within the article and supplementary materials.

## Supplementary Materials

The following supporting information can be downloaded at https://www.mdpi.com/article/10.3390/genes14112071/s1, Table S1: Correction factors for LDL-C on statins and ezetimibe treatment. Table S2: Clinical characteristics of adult probands and all adult individuals. Table S3: Clinical profiles and pretreatment lipid levels of positive and negative adult probands. Table S4: Clinical profiles and pretreatment lipid levels of all pediatric individuals. Table S5: Clinical profiles and pretreatment lipid levels of positive and negative pediatric probands.
