# Supplementary material for: Genetic Spectrum and Cascade Screening of Familial Hypercholesterolemia in Routine Clinical Setting in Hong Kong
Source: Genes (Basel). 2023 Nov 13;14(11):2071. doi: 10.3390/genes14112071 (PMC10671696; doi:10.3390/genes14112071)
Supplement: Supplementary file 1 [file genes-14-02071-s001.zip › genes-2719858-supplementary.pdf]

Table S1. Correction factors for LDL-C on statins and ezetimibe treatment.

Statin Dose (mg)	Correction factor	Add on	Correction factor
Simvastatin			
10	1.4		1.9
20	1.6		2.0
40	1.7		2.3
80	1.9		2.4
Atorvastatin			
10	1.6		2.0
20	1.8	+ Ezetimibe 10 mg	2.2
40	2.0		2.2
80	2.2		2.5
Rosuvastatin			
5	1.8		/
10	1.9		2.5
20	2.1		2.7
40	2.4		3.3

For unavailable pretreatment LDL-C, an estimate was obtained by multiplying the measured LDL-C by the appropriate factor.

Table S2. Clinical characteristics of adult probands and all adult individuals

A) Clinical profiles and pretreatment lipid levels of adult probands and all adult individuals

Characteristics	Probands (n=19)	All cases (n=31)
Gender	M: 11 + F:8	M: 26 + F: 15
Age (years)*	52.5 +/- 14.3	50.8 +/- 13.7
Consanguinity	0.0%	0.0%
Age of Hyperlipidemia diagnosed (years)*	35.9 +/- 14.6	36.6 +/- 13.6
Age of Genetic testing taken (years)*	50.0 +/- 13.8	48.1 +/- 13.4
Time gap in between (years)*	13 (18)	20 (20)
Having family history of		
1) Hyperlipidemia in first degree relatives	94.7%	93.5%
2) Hyperlipidemia in any degree relatives	94.7%	93.5%
3) pCVD	63.2%	58.1%
4) Stroke	10.5%	29.0%
5) Sudden death	10.5%	6.5%
Having past medical history		
1) Smoker	15.8%	9.7%
2) Hypertension	26.3%	25.8%
3) Diabetes mellitus	15.8%	19.4%
4) Ischemic heart disease	26.3%	16.1%
5) Stroke	0.0%	0.0%
6) Overweight/ Obesity	57.9%	51.6%
Having physical sign		
1) Xanthoma/ Xanthelasma	21.1%	12.9%
2) Arcus cornealis	5.3%	3.2%
Pretreatment lipid level (mmol/L)*		
1) Total triglyceride	1.74 +/- 0.98	1.70 +/- 0.92
2) Total cholesterol	10.28 +/- 2.48	9.30 +/- 2.50
3) HDL-C	1.72 +/- 0.99	1.50 +/- 0.80
4) LDL-C	8.42 +/- 2.37	7.56 +/- 2.47
Peak lipid level (mmol/L)*		
1) Total cholesterol	10.82 +/- 3.45	9.53 +/- 3.41
2) LDL-C	9.44 +/- 2.56	8.03 +/- 2.86
Criteria		
1) DLCNC score*	9.4 +/- 3.2	7.8 +/- 3.7

	Definite FH (> 8): 68.4%<br>Probable FH (6-8): 21.1%<br>Possible FH (3-5): 10.5%<br>FH +ve: 100.0%	Definite FH (> 8): 48.4%<br>Probable FH (6-8): 29.0%<br>Possible FH (3-5): 9.7%<br>FH +ve: 87.1%
2) modified DLCNC score*	10.5 +/- 2.5<br>Definite FH (> 8): 89.5%<br>Probable FH (6-8): 10.5%<br>Possible FH (3-5): 0.0%<br>FH +ve: 100.0%	9.3 +/- 2.9<br>Definite FH (> 8): 77.4%<br>Probable FH (6-8): 6.5%<br>Possible FH (3-5): 16.1%<br>FH +ve: 100.0%
Proportion of individual meeting FH diagnosis according to:		
3) Simon Broome Register	Definite FH: 21.1%<br>Possible FH: 73.7%	Definite FH: 67.7%<br>Possible FH: 12.9%
4) MEDPED	89.5%	80.6%
5) JFHMC	94.7%	80.6%
6) HK recommendation	100.0%	87.1%
7) HK recommendation of LDL-C 5.5 mmol/L	84.2%	74.2%
Treatment		
1) Treatment intensity*	2.32 +/- 0.90	2.26 +/- 0.90
2) Currently on statin	100%	90.3%
3) Currently on PCSK9 inhibitor	21.1%	13.3%
4) Percentage of LDL-C drop (%)*	59.2 +/- 22.5	55.0 +/- 23.5
5) Achieve LDL-C of < 2.5 mmol/L	31.6%	32.3%
6) Achieve LDL-C of < 1.8 mmol/L	10.5%	12.9%
*Data are presented as mean +/- SD, except for time gap in between which is presented as median (IQR)		

B) Clinical characteristics based on genetic diagnosis in adult cohort (probands and cascade screening cases included)

Types of variants	Total number	Peak LDL-C (mmol/L)	Treatment Intensity	Offer PCSK9 inhibitor	Percentage of LDL-C drop (%)	Achieve LDL-C of <2.5 mmol/L	Achieve LDL-C of <1.8 mmol/L
Single variant	14	9.05 +/- 2.25	2.56 +/- 0.39	35.7%	64.7 +/- 13.7	28.6%	14.3%
LDLR (total)	14	9.05 +/- 2.25					
Receptor-negative	5	8.96 +/- 2.15	2.48 +/- 0.53	20.0%	59.2 +/- 15.4	20.0%	0.0%
Splicing	3	7.87 +/- 2.11					
Frameshift	1	11.21					
Nonsense	1	10					
Receptor-defective	9	9.10 +/- 2.44	2.60 +/- 0.32	44.4%	67.8 +/- 12.6	33.3%	22.2%

Missense	9	9.10 +/- 2.44					
Two variants	2	10.60 +/- 1.10	2.83 +/- 0.67	100.0%	33.5 +/- 67.8	50.0%	0.0%
LDLR + LDLR	2	10.60 +/- 1.10					
*Data are presented as mean +/- SD if more than 1 datum							

Table S3. Clinical profiles and pretreatment lipid levels of positive and negative adult probands

Characteristics	Negative (n=3)	Positive (n=16)	p-value
Age (years)*	53.7 +/- 12.5	52.3 +/- 15.0	0.9
Consanguinity	0.0%	0.0%	1.0
Age of Hyperlipidemia diagnosed (years)*	42.0 +/- 7.9	34.8 +/- 15.5	0.5
Age of Genetic testing taken (years)*	51.3 +/- 13.7	49.8 +/- 14.2	0.9
Time gap in between (years)*	9 (19)	14.5 (20.5)	0.5
Having family history of			
1) Hyperlipidemia in first degree relatives	100.0%	93.8%	1.0
2) Hyperlipidemia in any degree relatives	100.0%	93.8%	1.0
3) pCVD	0.0%	75.0%	0.036*
4) Stroke	0.0%	12.5%	1.0
5) Sudden death	0.0%	12.5%	1.0
Having past medical history			
1) Smoker	0.0%	18.8%	1.0
2) Hypertension	33.3%	25.0%	1.0
3) Diabetes mellitus	0.0%	18.8%	1.0
4) Ischemic heart disease	0.0%	31.3%	0.5
5) Stroke	0.0%	0.0%	1.0
6) Overweight/ Obesity	66.7%	56.3%	1.0
Having physical sign			
1) Xanthoma/ Xanthelasma	0.0%	25.0%	1.0
2) Arcus cornealis	0.0%	6.3%	1.0
Pretreatment lipid level (mmol/L)*			
1) Total triglyceride	1.48 +/- 0.87	1.81 +/- 1.04	0.6
2) Total cholesterol	10.00 +/- 3.29	10.35 +/- 2.41	0.8
3) HDL-C	2.77 +/- 1.55	1.33 +/- 0.26	0.2
4) LDL-C	6.53 +/- 1.74	8.77 +/- 2.35	0.1
Peak lipid level (mmol/L)*			
1) Total cholesterol	14.17 +/- 5.35	10.19 +/- 2.79	0.2
2) LDL-C	10.50 +/- 2.45	9.24 +/- 2.27	0.2
Criteria			
1) DLCNC score*	7.3 +/- 2.9	9.8 +/- 3.2	0.3
2) modified DLCNC score*	9.0 +/- 0	10.8 +/- 2.7	0.3
Proportion of individual meeting FH diagnosis according to:			
3) Simon Broome Register	Definite FH: 0.0%<br>Possible FH: 100.0%	Definite FH: 25.0%<br>Possible FH: 68.8%	1.0
4) MEDPED	100.0%	87.5%	1.0
5) JFHMC	100.0%	93.8%	1.0

6) Hong Kong guideline	100.0%	100.0%	1.0
7) Hong Kong recommendation of LDL-C 5.5	66.7%	87.5%	0.5
Treatment			
1) Treatment intensity*	0.90 +/- 1.56	2.59 +/- 0.41	0.1
2) Currently on statin	100%	100%	1.0
3) Currently on PCSK9 inhibitor	0.0%	25.0%	1.0
4) Percentage of LDL-C drop (%)*	50.7 +/- 6.7	60.8 +/- 24.1	0.1
5) Achieve LDL-C of < 2.5 mmol/L	33.3%	31.3%	1.0
6) Achieve LDL-C of < 1.8 mmol/L	0.0%	12.5%	1.0
*Data are presented as mean +/- SD, except for time gap in between which is presented as median (IQR)			

Table S4. Clinical profiles and pretreatment lipid levels of all pediatric individuals

Characteristics	Probands (n=12)	Individual undergoing cascade screening (n=3)	Total (n=15)
Age (years)*	10.0 +/- 5.1	8.4 +/- 3.1	9.6 +/- 4.8
Consanguinity	0.0%	0.0%	0.0%
Age of Hyperlipidemia diagnosed (years)*	8.7 +/- 4.0	8.4 +/- 3.1	8.6 +/- 3.9
Age of diagnosis of FH (years)*	10.1 +/- 5.2	8.4 +/- 3.1	9.8 +/- 4.9
Having family history of			
1) Hyperlipidemia in first degree relatives	91.7%	100.0%	93.3%
2) Hyperlipidemia in any degree relatives	100.0%	100.0%	100%
3) pCVD	41.7%	66.7%	46.7%
4) Stroke	25.0%	0.0%	20%
Having physical sign			
1) Xanthoma/ Xanthelasma	16.7%	0.0%	13.3%
2) Arcus cornealis	0.0%	0.0%	0.0%
Pretreatment lipid level (mmol/L)*			
1) LDL-C	7.03 +/- 4.24	6.30 +/- 0.51	6.88 +/- 3.81
Criteria			
1) DLCNC score*	5.0 +/- 4.9	5.0 +/- 1.4	5.0 +/- 4.4
Proportion of individual meeting FH diagnosis according to:			
2) Simon Broome Register	Definite FH: 16.7%<br>Possible FH: 83.3%	Definite FH: 33.3%<br>Possible FH: 66.7%	Definite FH: 20.0%<br>Possible FH: 80.0%
3) MEDPED	100.0%	100.0%	100.0%
4) JFHMC	100.0%	100.0%	100.0%
5) Hong Kong guideline	100.0%	100.0%	100.0%
*Data are presented as mean +/- SD			

Table S5. Clinical profiles and pretreatment lipid levels of positive and negative pediatric probands

Characteristics	Negative (n=6)	Positive (n=6)
Age (years)*	11.7 +/- 3.5	8.2 +/- 5.7
Consanguinity	0.0%	0.0%
Age of Hyperlipidemia diagnosed (years)*	9.7 +/- 2.4	7.7 +/- 5.0
Age of diagnosis of FH (years)*	12.0 +/- 3.9	8.3 +/- 5.7
Having family history of		
1) Hyperlipidemia in first degree relatives	83.3%	100.0%
2) Hyperlipidemia in any degree relatives	100.0%	100.0%
3) pCVD	16.7%	66.7%
4) Stroke	16.7%	33.3%
Having physical sign		
1) Xanthoma/ Xanthelasma	0.0%	33.3%
2) Arcus cornealis	0.0%	0.0%
Pretreatment lipid level (mmol/L)*		
1) LDL-C	4.70 +/- 0.22	9.36 +/- 5.00
Criteria		
1) DLCNC score*	1.8 +/- 0.4	8.2 +/- 5.3
Proportion of individual meeting FH diagnosis according to:		
2) Simon Broome Register	Definite FH: 0.0%<br>Possible FH: 100.0%	Definite FH: 33.3%<br>Possible FH: 66.7%
3) MEDPED	100.0%	100.0%
4) JFHMC	100.0%	100.0%
5) Hong Kong guideline	100.0%	100.0%
*Data are presented as mean +/- SD		
